# Identification of swine influenza virus epitopes and analysis of multiple specificities expressed by cytotoxic T cell subsets

**DOI:** 10.1186/1743-422X-11-163

**Published:** 2014-09-06

**Authors:** Lasse E Pedersen, Solvej Ø Breum, Ulla Riber, Lars E Larsen, Gregers Jungersen

**Affiliations:** National Veterinary Institute, Technical University of Denmark, Frederiksberg C, Denmark

**Keywords:** Swine influenza virus, Major histocompatibility complex, Cytotoxic T cell, Viral epitope

## Abstract

**Background:**

Major histocompatibility complex (MHC) class I peptide binding and presentation are essential for antigen-specific activation of cytotoxic T lymphocytes (CTLs) and swine MHC class I molecules, also termed swine leukocyte antigens (SLA), thus play a crucial role in the process that leads to elimination of viruses such as swine influenza virus (SwIV). This study describes the identification of SLA-presented peptide epitopes that are targets for a swine CTL response, and further analyses multiple specificities expressed by SwIV activated CTL subsets.

**Findings:**

Four SwIV derived peptides were identified as T cell epitopes using fluorescent influenza:SLA tetramers. In addition, multiple CTL specificities were analyzed using peptide sequence substitutions in two of the four epitope candidates analyzed. Interestingly both conserved and substituted peptides were found to stain the CD4^-^CD8^+^ T cell subsets indicating multiple specificities.

**Conclusions:**

This study describes a timely and cost-effective approach for viral epitope identification in livestock animals. Analysis of T cell subsets showed multiple specificities suggesting SLA-bound epitope recognition of different conformations.

## Background

During the last two centuries influenza virus has constantly challenged animal and human health by seasonal outbreaks, most recently illustrated in the emerging 2009 pandemic H1N1 virus which, according to the World Health Organization (WHO), lead to more than 18.000 human deaths. Swine influenza virus (SwIV) is a common pathogen involved in the porcine respiratory disease complex. Beyond the veterinary implications, influenza infections in pigs also imply an important public health risk due to potential inter-species transmission of new reassortant strains of influenza viruses with pandemic capacity [1–4]. Human influenza virus vaccines are regularly updated with contemporary strains in contrast to commercially SwIV vaccines leading to inadequate protection against antigenic diverse viruses. In order to address new vaccine approaches which, based on common T cell epitopes, are able to provide a broader protection against a range of antigenic different viral strains, it is necessary to identify the peptide epitopes that are targets for a swine cytotoxic T cell response.

The selective binding and presentation of peptides in MHC complexes play a crucial role in the adaptive immune response to infectious diseases and vaccines [5, 6]. Such peptide:MHC (pMHC) complexes are scanned by circulating CD4^-^CD8^+^ cytotoxic T cells (CTLs) of the host immune system, occasionally leading to immune activation if the peptide is of foreign origin representing a potential danger to the host. To date pMHC tetramers have been described in work related to the analysis of mice [7], human [8, 9], bovine [10], and porcine [11] immune responses. In pigs, MHC class I molecules are termed swine leukocyte antigens (SLA) and one of the most commonly occurring SLA alleles, the SLA-1*0401 [12], has recently been mapped for its peptide binding preferences [13]. This study illustrates the use of pSLA fluorescent tetramers to identify SwIV derived epitopes. In summary, porcine fluorescent tetramers were generated with carefully selected influenza virus peptide ligands to measure immune responses against swine influenza virus after immunization of SLA class I matched pigs with inactivated virus.

## Methods

A total of 20 pigs were used in this study of which 16 expressed the SLA-1*0401 class I molecule. All procedures of animal handling and experimentation were approved by the Danish Animal Experiments Inspectorate. Experimental animals received chemically (C_3_H_4_O_2_) inactivated swine influenza A virus of different strains given in equal volumes of *Freund’s Incomplete* adjuvant with 4 repeated immunizations at three-week intervals (Table 1). Initially, blood samples were collected from all pigs followed by SLA allele typing using PCR-SSP [14–16]. Candidate SwIV epitopes were selected using *in silico* predictions for binding by the online available *NetMHCpan* algorithm [17–19], and combined with previously mapped preferences expressed by SLA-1*0401 [13]. Chosen candidate epitopes were then tested for SLA-1*0401 binding affinity using a previously described immunosorbent assay [20]. pSLA-1*0401 based fluorescent tetramers were produced as described previously [9], and porcine CD8^+^ cytotoxic T cell labeling was analyzed by flow cytometry. APC- and BV_421_-fluorochromes were used for labeling tetramers whereas PE-conjugated mAb against porcine CD8α (clone 76-2-11, BD Pharmingen) and FITC-conjugated mAb against porcine CD4 (clone 74-12-4, BD Pharmingen) were used for additional cell surface staining.

**Table 1:** **Influenza peptide epitopes and immunization strains**

SLA tetramer peptide epitopes				Immunization strains 1-5				
				1	2	3	4	5
SwIV candidate epitope	Viral protein of origin	AA position* in virus	Nucleotide position* in virus	A/swine/Den mark/101310- 1/2011(H1N1pdm09)	A/swine/Denmark/101568-1/2011(H1pdmN2†)	A/swine/Denmark/19126/1993 (H1N1)	A/swine/Denmark/101490-3/2011(H1N1)	A/swine/Denmark/1037-2/2011(H1N2†)
CTELKLSDY	NP	44-52	130-156	+	+	CTEL**Q**LSDY	CTEL**Q**LSDY	CTEL**Q**LSDY
GTEKLTITY	PB2	623-531	1567-1593	+	+	+	+	+
SSSFSFGGF	PB2	320-328	958-984	+	+	+	+	+
YVFVGTSRY	HA	215-223	643-669	+	+	YV**S**V**ES**SKY	YV**S**V**VS**SKY	YV**S**V**VS**SKY

Comparison of influenza virus candidate epitope sequences within the different viral strains used for immunizations. (+) SwIV candidate epitope sequence is 100% conserved in the viral strain used for immunization. (*) Amino acid position relative to start codon in virus A/swine/Denmark/12687/2003, (†) reassortant swine influenza virus encoding a human-like N2 gene [21]. Amino acids in bold mark substitutions in the sequence within the immunization strain compared to the respective candidate epitopes used for tetramer analysis.

## Results

**Figure 1:**
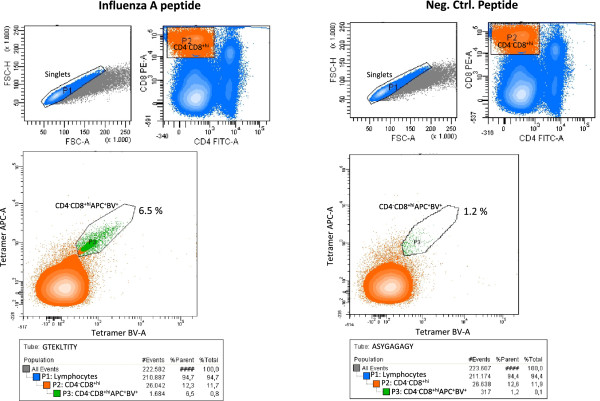
**Influenza virus tetramer staining of porcine CD4** ^**-**^ **CD8** **α** ^**high**^ **T cells.** SwIV tetramer staining of CD4^-^CD8α^high^ T cell subsets. Individual samples were stained by an epitope candidate tetramer (GTEKLTITY) and a negative control tetramer (ASYGAGAGY). Singlet lymphocytes are gated in P1 (blue). CD4^-^CD8α^high^ cells are gated in P2 (orange), and CD4^-^CD8α^high^ APC^+^BV^+^ tetramer double positive cells are shown in P3 (green) for animal number 4. Percentages of tetramer reactive cells within the CD4^-^CD8α^high^ population are shown for each sample.

Virally derived T cell epitopes in swine were identified by *ex vivo* analysis of candidate epitope peptides, based on *in silico* predictions and *in vitro* validation. Four influenza virus derived candidate epitope peptides (CTELKLSDY, GTEKLTITY, SSSFSFGGF, YVFVGTSRY) and one synthetically designed reference peptide (ASYGAGAGY) were selected for analysis based on a prediction to be bound by the SLA-1*0401 molecule. All selected peptides had *NetMHCpan* prediction rank scores of 1.00 or lower meaning that the peptide had a predicted affinity within the 1 percentile best candidates compared to a pool of 1,000.000 natural peptides (Table 2) [17–19]. Following *in vitro* testing it was found that all four influenza virus peptides were bound with high affinity by the SLA-1*0401 MHC class I molecule, and identified as T cell epitopes by *ex vivo* flow cytometry analysis using influenza:SLA tetramers. Positive samples were defined by a minimum threshold of 2-fold higher staining percentage compared to the negative background control, as previously set by others [22]. Six of the 16 SLA-matched pigs were found to express activated CTL populations showing specificities against the SwIV peptides post immunization (Table 3). SwIV tetramer staining above the 2-fold threshold ranged between 0.8 and 5.3% of the total CD4^-^CD8α^high^ cell population depending on the different epitopes and animals (Table 3, bold numbers). A specific T cell subset of 6.5% of the CD4^-^CD8α^high^ population stained positive for the GTEKLTITY epitope as compared to the negative background control of 1.2% (Figure 1). In addition, substitutions were introduced in 50% of the epitope candidates to examine individual T cell subsets in regard to the expression of multiple T cell receptor (TCR) specificities. Interestingly both conserved and substituted epitope candidates were found to stain the CD4^-^CD8α^high^ T cell subsets. Staining percentages of epitopes including amino acid substitutions compared to their respective immunization strain are marked by an asterix (Table 3).

**Table 2:** **Peptide predictions and affinities**

Peptide sequence	***NetMHCpan***prediction rank	SLA-1*0401 affinity K _D_(nM)
**CTELKLSDY**	1.00	16
**GTEKLTITY**	0.80	34
**SSSFSFGGF**	0.80	378
**YVFVGTSRY**	0.10	325
**ASYGAGAGY**	0.05	19

Peptide sequences selected for affinity analysis based on *NetMHCpan* prediction ranks and SLA-1*0401 amino acid requirements for binding. The lower the K_D_ value the higher the affinity for binding. Peptides having K_D_ values <500 nM are considered as intermediate affinity ligands whereas a K_D_ value <100 nM represents a high affinity binding peptide ligand.

**Table 3:** **Influenza virus tetramer staining**

Animal ID/SwIV strain	Tetramer SwIV peptide	Peptide substituted from immunization strain	Frequency of tetramer (APC + BV421+) cells
			(Tetramer + cells subtracted negative control)
	ASYGAGAGY	Negative control	0.80 (*0.00*)
**1/**	CTELKLSDY	No	1.70 (**0.90**)
**1**	GTEKLTITY	No	1.90 (**1.10**)
	SSSFSFGGF	No	1.70 (**0.90**)
	YVFVGTSRY	No	1.60 (**0.80**)
	ASYGAGAGY	Negative control	0.60 (*0.00*)
**2/**	CTELKLSDY	No	1.70 (**1.10**)
**3**	GTEKLTITY	No	1.50 (**0.90**)
	SSSFSFGGF	No	1.40 (**0.80**)
	YVFVGTSRY	No	1.50 (**0.90**)
	ASYGAGAGY	Negative control	1.20 (*0.00*)
	CTELKLSDY	No	6.30 (**5.10**)
**4/**	GTEKLTITY	No	6.50 (**5.30**)
**3**	SSSFSFGGF	No	3.90 (**2.70**)
	YVFVGTSRY	No	5.80 (**4.60**)
	ASYGAGAGY	Negative control	2.60 (*0.00*)
	CTELKLSDY	Yes	5.80 (3.20*)
**6/**	GTEKLTITY	No	5.80 (**3.20**)
**3**	SSSFSFGGF	No	4.90 (*2.30*)
	YVFVGTSRY	Yes	5.90 (3.30*)
	ASYGAGAGY	Negative control	0.90 (*0.00*)
	CTELKLSDY	Yes	3.00 (2.10*)
**8/**	GTEKLTITY	No	2.40 (**1.50**)
**4**	SSSFSFGGF	No	1.90 (**1.00**)
	YVFVGTSRY	Yes	2.70 (1.80*)
	ASYGAGAGY	Negative control	1.10 (*0.00*)
	CTELKLSDY	Yes	2.80 (1.70*)
**16/**	GTEKLTITY	No	2.50 (**1.40**)
**5**	SSSFSFGGF	No	2.30 (**1.20**)
	YVFVGTSRY	Yes	2.70 (1.60*)

Tetramer staining frequencies. Percentile numbers in bold show specific tetramer staining post background subtraction. The relative background staining has been defined for each animal by a negative control tetramer (ASYGAGAGY). Italic percentile numbers indicate non-specific staining. Percentages marked by an asterix (*) indentify positive staining by influenza peptides which are sequence substituted compared to the respective immunization strains.

## Discussion and conclusion

This study describes a timely and cost-effective approach for viral epitope analysis and identification in livestock animals. In addition, we hypothesized CD8^+^ cytotoxic T cell subsets to possess multiple specificities. Interestingly, it was found that conserved as well as substituted epitopes positively stained T cell subsets suggesting SLA-bound epitope recognition of different conformations. These findings correlate with previous studies showing that CTL subsets expressing individual TCRs are capable of recognizing ligands of various conformations presented by the same MHC [23, 24].

In conclusion, the data and approaches described have great potential for future studies using the pig as a large animal model for viral epitope identification. Furthermore, by including sequence substituted MHC ligands in the analysis it was illustrated how CD4^-^CD8^+^ T cell subsets were capable of expressing multiple T cell receptor ligand specificities. Finally, identification of T cell epitopes conserved across all types, subtypes and strains of influenza viruses, and including mutations, can be valuable knowledge in terms of future vaccine design as well as in achieving a better understanding of the immune responses elicited by vaccination and natural infection.
